# Polymers Use as Mulch Films in Agriculture—A Review of History, Problems and Current Trends

**DOI:** 10.3390/polym14235062

**Published:** 2022-11-22

**Authors:** Zinnia Mansoor, Fideline Tchuenbou-Magaia, Marek Kowalczuk, Grazyna Adamus, Georgina Manning, Mattia Parati, Iza Radecka, Habib Khan

**Affiliations:** 1 School of Sciences, Faculty of Science and Engineering, University of Wolverhampton, Wolverhampton WV1 1LY, UK; 2 Department of Biotechnology, Virtual University of Pakistan, Lahore 54000, Pakistan; 3 Division of Chemical Engineering, School of Engineering, Computing and Mathematical Sciences, Faculty of Science and Engineering, University of Wolverhampton, Wolverhampton WV1 1LY, UK; 4 Centre of Polymer and Carbon Materials, Polish Academy of Sciences, M. Curie-Sklodowskiej 34, 41-819 Zabrze, Poland

**Keywords:** mulch films, biodegradability, biopolymers, SDGs, plastic pollution

## Abstract

The application of mulch films for preserving soil moisture and preventing weed growth has been a part of agricultural practice for decades. Different materials have been used as mulch films, but polyethylene plastic has been considered most effective due to its excellent mechanical strength, low cost and ability to act as a barrier for sunlight and water. However, its use carries a risk of plastic pollution and health hazards, hence new laws have been passed to replace it completely with other materials over the next few years. Research to find out about new biodegradable polymers for this purpose has gained impetus in the past few years, driven by regulations and the United Nations Organization’s Sustainable Development Goals. The primary requisite for these polymers is biodegradability under natural climatic conditions without the production of any toxic residual compounds. Therefore, biodegradable polymers developed from fossil fuels, microorganisms, animals and plants are viable options for using as mulching material. However, the solution is not as simple since each polymer has different mechanical properties and a compromise has to be made in terms of strength, cost and biodegradability of the polymer for its use as mulch film. This review discusses the history of mulching materials, the gradual evolution in the choice of materials, the process of biodegradation of mulch films, the regulations passed regarding material to be used, types of polymers that can be explored as potential mulch films and the future prospects in the area.

## 1. Introduction

The technique of mulching has been a part of agricultural practice for a long time. In simple terms, mulches are defined as materials that are applied directly onto the surface of soil for various purposes such as the protection of seedlings and young shoots through insulation, reduction of evaporation, control of weed growth and prevention of soil erosion [1]. They specifically protect delicate crop species from unfavourable abiotic and biotic stresses that may occur as a result of extreme weather conditions, insects, pests and weeds. Therefore, mulches are commonly used in agriculture to prevent loss of crop yield [2].

**Figure 1:**
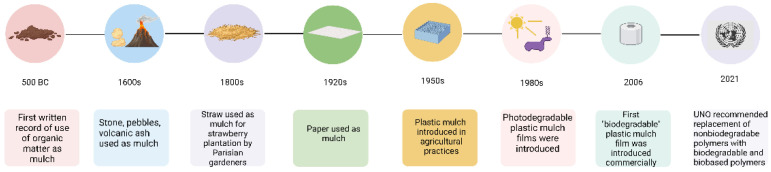
The history and development of different mulching materials.

The history of using mulching to enhance crop production dates back to around 500 BCE, as shown in Figure 1. It is from that age that the first documented proof of the use of organic matter as a mulch film has been obtained [3]. The material used gradually changed to stones, pebbles and volcanic ash in the 1600s, although these were mostly used in arid regions. In the 1800s the Parisian market gardeners found that the use of straw as mulching material for strawberry production was beneficial. Thus, over a span of hundreds of years, different naturally available materials were tried and used for mulching depending upon climatic conditions in different parts of the world [4]. As science advanced in the 20th century, mulching was also revolutionized. Paper sheets were introduced as mulch films in the 1920s, followed by the commercialization of plastic, specifically polyethylene films for mulching in the late 1950s. Plastic mulching gained popularity and proved to be very effective. However, the negative impact of plastic became evident within three decades and by the early 1980s photo-degradable and oxo-degradable plastics were introduced as an alternative to polyethylene based films. It soon became apparent that these polymers did not degrade in field conditions and generated microplastics [5]. Research was accelerated in this area and in 2006 the first biodegradable plastic mulch film was introduced in the market commercially. Following this, a number of biodegradable mulch films were manufactured by companies throughout the world. It was not until 2021, however, that the Food and Agriculture Organization, which is a part of the United Nations Organization, gave its recommendation to replace conventional and non-biodegradable polymers with bio-based and biodegradable materials for mulching practices [6].

Various anthropogenic activities over the years have led to the production of greenhouse gases, which, in turn, have resulted in climate change. The emission and accumulation of these gases in the atmosphere has led to global warming, i.e., an increase in average temperatures and fluctuations that contribute to extreme weather conditions, as well as alterations in precipitation patterns throughout the world. Such changes in agricultural regions have an adverse impact on crop production, thus creating problems when providing adequate food supply for an increasing population in an efficient and sustainable manner [7]. The limited availability of arable land, depletion of water sources for irrigation, soil erosion, overexploitation of natural resources, pollution of ecosystems and climate change are some of the factors that restrict food crop production and yields [8].

**Figure 2:**
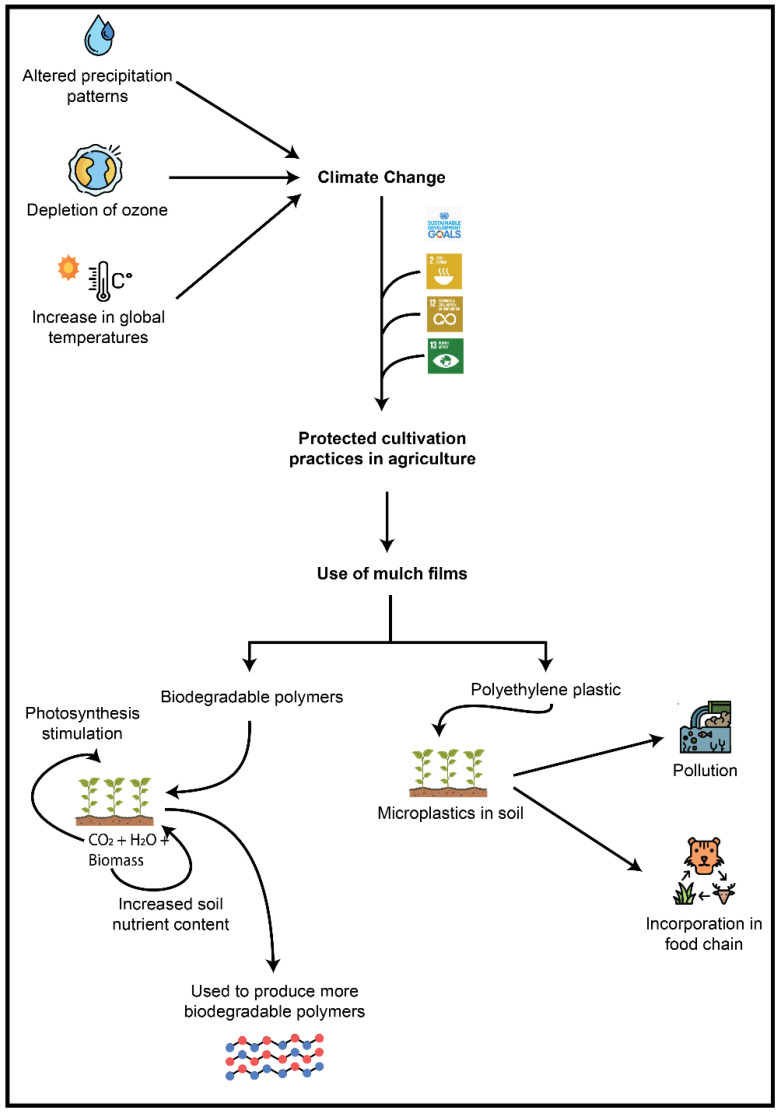
The need for mulching as an agricultural practice and the materials available for use as mulch films.

In 2015, under the banner of the United Nations Organization, the international community developed a series of Sustainable Development Goals, known as SDGs, which include ensuring access to food for all, increasing agricultural productivity and achieving zero hunger by 2030 [9]. These goals not only imply the provision of food to achieve zero hunger on a global scale, but also aim to enhance agricultural productivity in a sustainable, manageable and efficient manner. The primary focus is to increase yields of food crops by adopting farming practices that are environmentally friendly and ecologically viable [10]. Mulching is already commonly used all over the world as a strategy to improve crop yield and prevent losses. The global mulch market is estimated at USD 3.5 billion in the year 2020, and is projected to amount to USD 5.1 billion by 2027 [11]. Considering the expansion of the market, it is pertinent to check that the practice is in line with the UN SDGs, specifically in terms of responsible production and consumption (Figure 2). Currently, the main material used for mulch films is polyethylene plastic, which is raising concerns. These mulch films do not degrade naturally and need to be removed from fields after harvesting. They cannot be recycled, and the debris left in soil contributes to soil pollution. A lack of disposal options for used plastic films adds to land and water pollution [12]. Therefore, there is an urgent need to explore other alternative polymers that may be used to replace plastic for mulching purposes [13].

Biodegradable polymers may be one solution, and have the potential to replace plastics for many applications. However, these polymers have certain limitations which are restricting their use on a commercial scale for the purpose of mulching. Factors that need to be considered before a polymer can be used include not only its physical and chemical properties but also the source and method of production. These polymers have different mechanical properties, which, in turn, affect their biodegradation in the environment [14]. Moreover, legislation and laws passed over the past few years have defined and strict criteria regarding biodegradation, chemical composition, deployment and toxicity for materials that may be used as a mulch film [15].

This review discusses the practice of mulching in agriculture, its benefits, the materials used as mulch films, and the alternate options that can be explored for sustainable agricultural practices. It also includes an overview of the biodegradation process that occurs in the soil for the breakdown of mulching materials, factors affecting this process, and a list of different commercially available mulches, highlighting the direction of further research in this area.

## 2. The Benefits of Mulch Films

The increasing food demand of a growing world population has driven the need to increase agricultural crop yields. Protected cultivation is one way to help optimize the yield of crops. This involves controlling the microclimate around the growing plant to protect it from harsh climatic conditions [16]. Mulching is based on the principle of protected cultivation and involves the application of a protective ground cover made of different materials that may be organic or synthetic, to improve the growth and yield of agricultural crops. The word mulch is derived from the German word ‘molsch’, which means ‘easy to decay’ [3].

**Figure 3:**
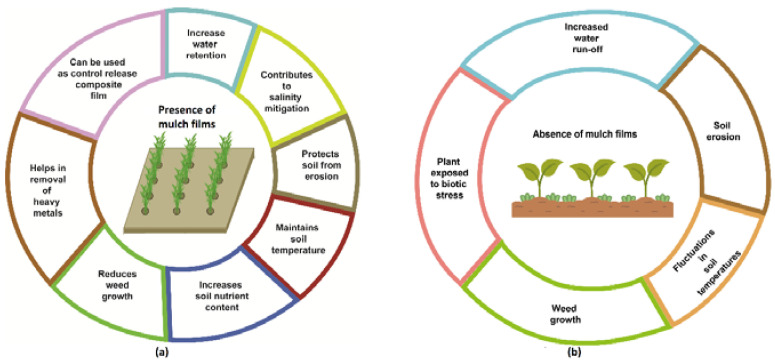
A comparison of the effect of mulch films on plant growth and soil—(**a**) in the presence of mulch films and (**b**) in the absence of mulch films.

The use of mulch films in agriculture has several benefits when compared to non-mulched crop production. These may be categorized as improvements in the soil microenvironment or economic advantages (Figure 3a). Soil moisture content is an important factor that affects the growth of plants. Winds, high temperature, adverse climatic conditions and particularly weed infestation can contribute to the reduction of soil moisture. Mulches have been reported to increase the percolation and water retention capacity of soil, to the extent that the use of mulching material can reduce the irrigation requirement of crop plants. This is attributed to their water retention ability, which reduces the runoff from the soil profile [17,18]. This can indirectly contribute to salinity mitigation, as well. Various studies have demonstrated that the application of mulch films reduces the impact of salt toxicity on plants and helps in soil reclamation [19]. Mulches are also associated with the protection of soil from wind and water erosion, as well as the reduction of the compaction of soil. It has been observed that crop growth is negatively affected due to erosion and soil compaction in the absence of mulch films (Figure 3b). Mulches protect the soil by breaking the speed of water, especially in slopes or hilly areas, thereby increasing the infiltration rate. Similarly, the presence of covering material in the form of mulch films prevents soil erosion by winds [20]. Mulch materials also reduce the impact of weathering and the beating action of heavy rain, and the weight of feet and tyres of heavy machinery, helping to overcome the problem of compaction in soil [21].

It is important to control the temperature of soil, since temperature fluctuations adversely affect the development of roots [20]. Since the material used for mulching covers the soil surface entirely, it helps to maintain the optimal soil temperature required for plant growth. Studies have shown that the application of mulch films keeps the soil temperature warmer on chilly days, and cooler during hot spells [17,22]. Mulches also contribute to the soil nutrient content, since these are broken down or degraded by soil microorganisms into simpler compounds that become a part of the soil itself [23]. Weed control is one of the key benefits that mulching provides. When mulch is spread on the surface of the soil, it acts as a barrier to the passage of sunlight, which reduces germination of weeds, especially small-seeded weed species. This phenomenon has been observed and widely used in nurseries as well as agricultural fields [24].

The presence of heavy metals in soil affects the growth of plants. Mulches can be used as a remediation strategy for the removal of these heavy metals from soil. Organic and plant-based mulch material forms complexes with heavy metals and converts them into a form that renders them non-toxic for plants [25]. Mulches can also be used as control release composite films, which are embedded with fertilizers, herbicides or pesticides. This strategy allows the gradual and slow release of these materials from the mulch films, ensuring that these are constantly available for use by the plant instead of being run off after the first application [26,27].

Generally, mulching decreases the stress level on plants, leading to better growth. This leads to enhanced crop yield and improved product quality. The crop may also be harvested earlier and tends to be more profitable [28]. All these facts confer economic advantages to the use of mulch films.

## 3. Types of Mulch Films

**Figure 4:**
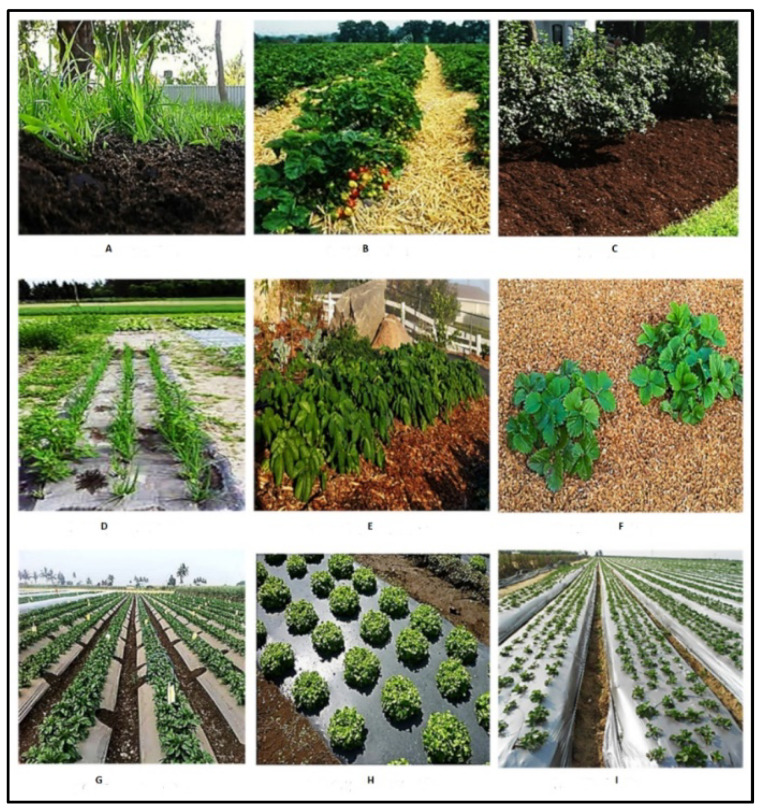
Application of different mulch films in the field—(**A**): Compost, (**B**): Straw, (**C**): Bark, (**D**): Newspaper, (**E**): Woodchips, (**F**): Sawdust, (**G**): Plastic, (**H**): Black plastic, (**I**): LDPE. Adapted from [30].

The use of mulch films dates back to ancient civilizations. The earliest mulch material comprised by-products from the agricultural and forestry industries, and included the trimmings of trees and shrubs, animal waste, stubble and residues of crop plants [20]. With time the material was modified, and now mulches can be categorized as organic or inorganic mulches. Organic mulches are made from materials found in nature and are usually broken down into simpler compounds by soil microorganisms. Inorganic mulches, on the other hand, are made of synthetic material that does not decompose easily [29]. The different types of materials used for mulching and their application in the field are shown in Figure 4.

The type of mulch used is governed by many factors, including the plant species, soil type and characteristics, the cost and availability of mulching material, as well as the regulations and law governing the region [31]. For instance, for vineyards or fruit orchards, the mulch film is generally thick and has a lifespan of years until it becomes ineffective. For vegetable fields, on the other hand, thinner mulch films are used that last one growing season only [32]. Similarly, the effect of each mulching material is a combination of various factors including the amount of material applied, the carbon and nitrogen ratio of the material, its thickness, colour and other physical attributes, and the amount of toxic substances present [33]. Each material has certain advantages and disadvantages regarding its use for mulching, as summarized in Table 1 [34,35,36,37,38,39,40,41,42,43,44,45,46,47,48,49,50,51,52,53,54,55,56,57,58,59,60,61,62,63,64,65,66,67,68,69,70,71,72].

### 3.1. Organic Mulches

Organic mulches mainly comprise animal or plant residues such as compost, manures, straws, husks, saw dust, grass and paper clippings, and wood/bark chips (Figure 4A–F). The application of compost and manure is an age-old practice in many regions of the world. The use of compost is as a way of recycling waste. It is cheap and readily available. Studies have exhibited that repeated use of compost for mulching over a number of years can increase the organic content of the soil and improve plant yield [34]. However, the use of compost as mulch has the added risk of phytotoxicity due to high nitrogen content. If applied near the stalks it absorbs moisture that promotes diseases and pests, leading to low crop yields [35]. Depending on the source of compost, it may contain heavy metals that accumulate in the soil as well as plants. While the presence of these heavy metals may not lead to toxicity in plants, it renders the crop unfit for human consumption. Compost is therefore not preferred anymore as a mulching material [36].

Other organic materials that can be used include straw and husks. These materials have a long life span when used for mulching and are effective for vegetables grown in winter months [37]. They are inexpensive, readily available and field trials have exhibited that they are good at preventing water loss via evaporation [38]. Both husk and straw mulches have the potential to increase crop yield and can significantly lower water losses from a well-irrigated system [39]. However, they often contaminate the soil with weed seeds and in addition harbour pests such as termites, snails, slugs and earwigs, leading to losses in crop yield, reducing their use for mulching [40].

Sawdust is readily available and has exhibited potential as a mulching material specifically for acid loving plants. It is very efficient in minimizing water runoff and soil erosion [41]. Studies have shown that it is not efficient in controlling the growth of weeds and also tends to harden over time, preventing water from reaching the deeper layers of the soil [42]. Another disadvantage is the low nitrogen content of sawdust, which means that it uses up nitrogen from the soil as it decomposes. This has been seen in field trials where the use of sawdust mulch reduced the number of flowers produced and had an adverse impact on plant growth [43].

Grass clippings are very effective as a mulching material when applied as an appropriately thick layer. If the layer is too thick, it prevents air from penetrating, resulting in rotten and odorous clippings. If the layer is thin, the grass clippings decompose quickly, and need to be replenished frequently to maintain efficacy of the mulching material [44]. Fresh grass clippings also have the potential to develop their own root systems and offer growth competition to the crop plants. The use of these has also been shown to increase soil temperature and affect plant growth negatively in hot climates, limiting their application as mulching material [45].

Paper mulches are made up of cellulose fibres which can be decomposed naturally by most soil-borne microorganisms. Sheets of paper, mainly newspapers with black ink, can be used as mulch, as a part of a recycling strategy. The colour of paper used for mulching has an impact on the control of weed growth. Black paper mulching has shown promising results for decreasing the growth of weeds in lettuce and cantaloupe farming [46]. However, the main problem is that due to their light weight, these clippings are easily blown away by the wind. When applied as sheets, they tear apart easily when damp or wet, and are penetrated by weeds. Paper mulches degrade quickly so they cannot be used for long-term cultivation. These disadvantages limit the use of paper as a mulching material [40,47].

Wood and bark chips are preferred as mulching materials since these are very effective in retaining moisture in the soil. They also allow proper aeration and contribute to the organic content of the soil. Pine bark makes an attractive, usually dark-coloured mulch and may be used for ornamental plants. However, bark chips and pine needles have been reported to reduce the soil pH upon application and cause phytotoxicity [48]. The decomposition of woody material results in the release of phenolic acids which contribute to acidification of the soil, affecting plant growth. While this may be beneficial for crops that require an acidic pH for growth, it limits the large-scale use of woody material for mulching [49].

The main factors that limit the use of organic mulches are that they may not be available in adequate amounts throughout the year, have an inconsistent quality and are extremely labour intensive [50].

### 3.2. Synthetic Mulches

Inorganic or synthetic mulches are made up of materials such as glass, rubber sheets and plastic [51]. Glass mulches are usually made from chips of bottled glass as a method of recycling parts that cannot be separated from organic matter and other debris. These are exquisitely used to aesthetically modify a landscape while functioning as a mulch film [1]. However, the use of glass mulch films has become limited over the years because of their low efficiency in controlling weed growth. The ability of glass to reflect light reduces the sunlight that can reach the soil and has an impact on the soil temperature [52]. Moreover, the incorporation of non-recyclable glass into soil disturbs the physico-chemical characteristics of the soil, which subsequently impairs plant growth [53].

Rubber mulches are frequently made by shredding worn out tyres as a strategy of recycling. These shredded tyres include very fine particulate matter that can be inhaled, ingested or taken up transdermally, increasing health risks. The use of rubber mulches is also associated with the hazard of ignition since rubber catches fire quickly and is very difficult to extinguish [54]. Moreover, it has also been reported that rubber mulches are less effective in controlling weed growth compared to organic mulches [55]. In fact, high levels of zinc incorporated in tyres during manufacturing is released in the soil from the mulch and leads to zinc toxicity in plants, which adversely affects their growth [56].

Plastic mulch films (Figure 4G–I) have been used extensively since the 1950s and their global market has shown a continuous growth with a worth of USD 4.1 billion a year [57]. These are very effective in controlling weed growth and over time they have increased global grain and cash crop yields by 15% and 40%, respectively [58]. Polyethylene plastic is the most popular and frequently used inorganic mulch throughout the world because of its efficacy and low price [59]. It is a low-density plastic that is highly resistant to weathering due to its chemical structure, which comprises saturated hydrocarbon chains [60]. These mulch films have low cost, low frequency of replacement, versatility and strength, and are therefore very effective in the field [61].

**Figure 5:**
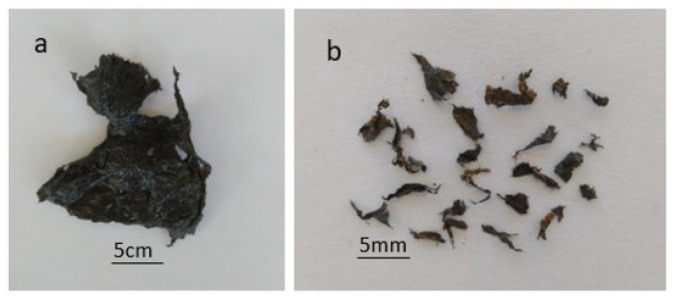
The debris of black plastic mulch films—(**a**): piece of plastic mulch film, (**b**): microplastics collected from soil after removal of mulch film. Adapted from [67].

However, there are several problems associated with the use of plastic mulch films, including laborious and costly removal from the fields after use, lack of sustainable end-of-life management options and the addition of plastic waste to the environment [62]. At the end of the growing season, the mulch film is often contaminated with plant as well as soil debris which restricts its recycling. Therefore, these are categorized as single-use plastic films and are usually landfilled or incinerated, contributing significantly to plastic pollution [63]. Complete removal of polyethylene mulch films is often not possible from fields, which has led to considerable amounts of macro and microplastic debris in soils as shown in Figure 5 [54]. This accumulation of plastic residue in soil has a negative impact on crop production by affecting the plant growth-promoting bacteria (PGPB), damaging soil structure, retarding root growth and development, and altering the carbon concentration of the soil [65]. In some countries the lack of disposal options means that farmers simply stockpile these mulch films after use, which subsequently leads to the dispersion of the mulch fragments into the environment by water and wind erosion, causing pollution [66].

Most countries in the world are now opting for sustainable practices and have banned single-use plastics to overcome plastic waste generation and pollution. This has necessitated the need to look for sustainable and eco-friendly alternatives that can be used as mulch films [68]. As a solution, pro-oxidant additive containing (PAC) plastics have been formulated. These are chemically similar to low density polyethylene plastics but contain a pro-oxidant additive that increases oxidation and degradation of the polymer in the presence of light [69]. This photo-oxidation reduces the molar mass and adds oxygenated groups that make the polymer more prone to breakdown by microorganisms under aerobic environments. These plastics are also referred to as ‘oxo-degradable’ plastics. An example is Oxo-PP or oxo-degradable polypropylene [70]. PAC plastic mulches have been reported to be as efficient as conventional plastic mulches in the field for controlling the growth of weeds. They also have no adverse impact on soil health [71].

However, these mulch films have low strength and can break down prematurely while being spread onto the field. This fragility and their lightweight nature makes the application very difficult. Moreover, the absence of UV light reduces degradation of these plastics once they are incorporated into the soil. Even if there is breakdown in the soil, it is never fully biodegraded. The mulch pieces persist in soil and watersheds, and form micro plastics that have the ability to adsorb toxic pesticides, insecticides and herbicides, and carry them into the food chain [3]. Research reports suggest that while these mulch films are more expensive than polyethylene plastic films, they contribute equally to the generation of micro plastics that pollute the soil [72]. Many countries in the European Union, including France, have completely banned the use of PAC plastics, making it imperative to explore more sustainable, economical and environment friendly alternatives [66].

## 4. Current Trends in Mulching Material

In recent years a lot of research has been carried out to produce mulch material that is both sustainable and eco-friendly. The material for a mulch film should ideally be bio-based and very prone to biodegradation [73]. To replace plastic, it should have similar mechanical properties including a high tensile strength and high percentage elongation. The European Union has set up the product standards for biodegradable mulch films that are to be used in the horticulture and agriculture sectors. The first and only standard (EN 17033) has specified values that must be met, in terms of biodegradability, deployment, chemical composition and eco-toxicity, for a material to be used as a mulch film [15]. The generally acceptable standard for biodegradability is that the material should completely breakdown to carbon dioxide, water, methane, inorganic compounds and biomass within a year of application without producing any visible, toxic residue [74].

**Figure 6:**
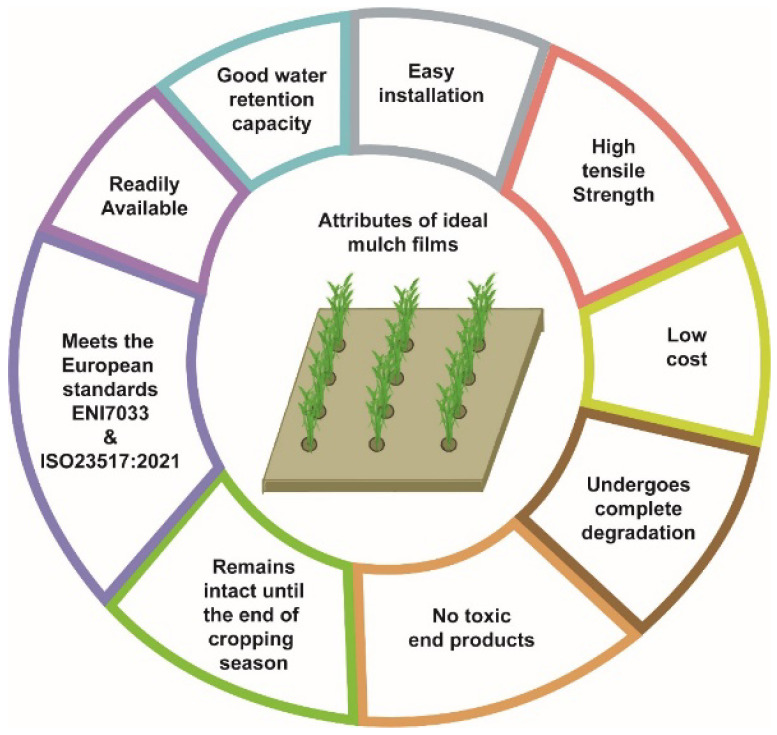
Properties of the ideal mulch film materials.

The use of biodegradable material imparts the main advantage of being tilled into soil once used, so that it can be degraded by the action of soil microbiota, reducing the costs of labour and disposal [75]. To replace conventional polyethylene mulch, the material should have the ability to maintain the optimum microclimate for plant growth and degrade completely without producing any harmful toxic products. In terms of efficacy, it should possess high tensile strength, good water retention capacity and allow for easy application. The film should be readily available, especially in the cropping season, and be inexpensive so that farmers can use it. Any material that is to be used as mulch should meet the minimum design requirements according to EN17033 and ISO 23517:2021 [76,77]. The main attributes required in a material to be used as mulch film are shown in Figure 6.

## 5. Biodegradable Polymers for Mulch Films

**Figure 7:**
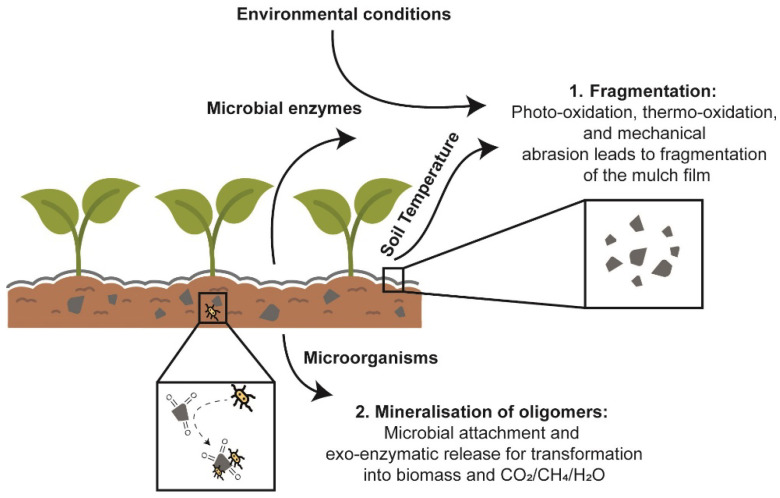
The two steps of biodegradation of mulch films.

The term biodegradation refers to the breakdown of macromolecules by the action of microorganisms. Molecular level studies have shown that it is a two-step process [78]. In the first step fragmentation occurs in which the high molecular weight polymer chain is broken down into smaller units that may be oligomeric units with polar chain ends or monomers with specific properties. Fragmentation may occur through hydrolysis, which may or may not be enzyme-mediated, oxidation or other chemical reactions depending on the environment as well as the chemical structure of the polymers. In the second step, oligomers and monomers formed in the first step are mineralized by microorganisms to produce carbon dioxide, methane, water, and biomass. The products formed may vary slightly depending upon the microorganisms involved and the aerobic/anaerobic nature of the process [79]. The schematic diagram of the process of degradation is shown in Figure 7.

Polymers may constitute aliphatic chains or aromatic groups. Generally aliphatic polymers are easily hydrolysable while aromatic polymers require harsh conditions for breakdown, such as extremely acidic environments at high temperatures. Most biodegradable polymers are therefore aliphatic, but some aliphatic-aromatic polymers that have a limited number of aromatic units may also be included in this category [80].

**Figure 8:**
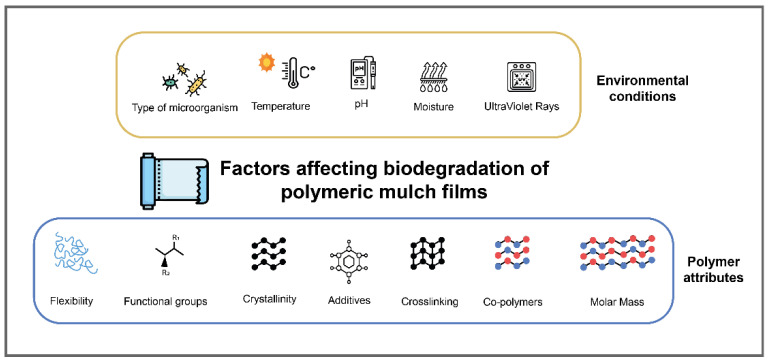
The factors that affect biodegradation of mulch films.

Many factors affect the biodegradation of a polymer, including environmental conditions and the characteristics of the polymer, as shown in Figure 8. Environmental conditions may further be categorized as abiotic or biotic factors [50]. Abiotic factors include temperature, moisture, pH, and the presence of UV radiation. It has been reported in numerous studies that the temperature of the soil has a significant effect on the initiation of the biodegradation process, and lower temperatures decrease the rate at which the polymer is broken down [81]. Soil moisture is important and may become a limiting factor when fragmentation proceeds through hydrolysis. It is less likely to slow down degradation during irrigation periods, but has a marked effect if the moisture content drops too low or increases to the extent of making the soil anoxic [74,82]. Soil pH has a marked effect on the metabolism of microorganisms which, in turn, has an impact on their ability to degrade mulch films. It has been observed that generally soils with a neutral pH show maximum biodegradation, although depending on the type of microorganisms present there may be exceptions [83]. Ultraviolet radiation has a direct impact on the breakdown of mulch films. Experiments have shown that UV radiation speeds up biodegradation two-fold [84]. Biotic factors that affect biodegradation include the type of microorganisms present, the enzymes produced by them, and their ability to colonize the surface of the mulch films to initiate biodegradation. Climate and soil characteristics define the type of microbial communities present indigenously. Whilst it is mostly bacteria that are involved in biodegradation, the presence of fungi may accelerate the process in some cases [85].

It is interesting to note that the biodegradability of a polymer is not linked to its source but is dependent on its physiochemical properties such as molar mass, cross-linking, functional groups, crystallinity, flexibility and the presence of co-polymers, additives or cross-linkers [86]. As a general rule, an increase in the molar mass leads to a decrease in degradation. This is also linked to the solubility of the polymer, since high molecular weight compounds have low solubility which makes them unfavourable for microbial attack [87]. Similarly, highly cross-linked polymers have slower degradation since the close mesh-like structure makes the polymer inaccessible to both microbes and water molecules. The polymer needs to be mechanically broken down into smaller pieces before biodegradation can occur [88]. The type of bonds and functional groups present in the polymer are directly linked to its degradation. The presence of hydrophobic and non-polar functional groups makes a polymer less prone to biodegradation [89]. The breakdown of a mulch film is dependent on the type of polymer present. The biodegradation mechanism becomes more complex if the film is made of more than one type of polymer. These co-polymer mulches or blends may be degraded at a slower or at times greater rate than single polymer mulches. For instance, it has been observed that PBAT blends degrade more slowly compared to PBAT mulches, whilst PLA blends show better degradation than mulches made of PLA alone [90,91]. Sometimes additives are included in polymer blends to increase the solubility or flexibility of the product. The presence of additives, specifically in co-polymers, may contribute to a reduction in its biodegradation. Depending on the type of additive, it may form cross-links with the polymer upon exposure to solar radiation. This cross-linking reduces accessibility to microbial enzymes and water, which leads to a decline in the degradation rate [92]. The degree of crystallinity of a polymer is one of the main factors that affect the biodegradation phenomenon. The more crystalline a polymer is, the denser the packing of molecules, which slows down the rate at which it can be broken down [93]. Although flexibility of a polymer seems more linked to its mechanical properties, it is surprising that it also has an impact on the rate of biodegradation. It has been observed that polymers with increased flexibility show enhanced biodegradation [94].

**Figure 9:**
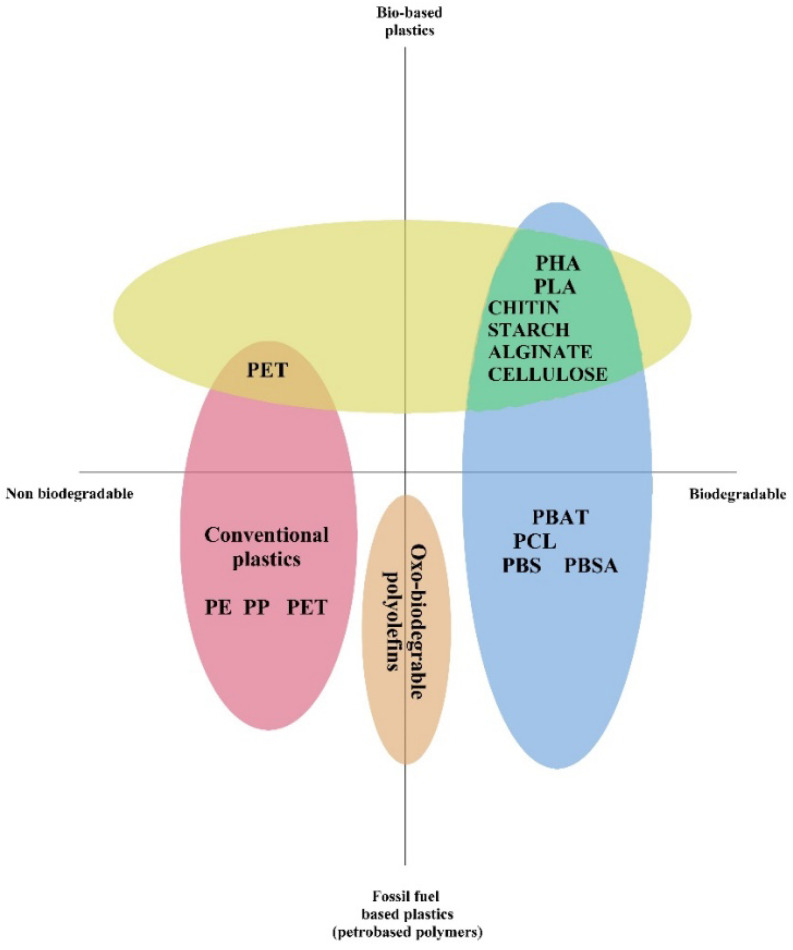
A comparison of the diversity of plastics and bio-based polymers. Abbreviations include: PBAT—Polybutyrate Adipate Terephthalate, PCL—Polycaprolactone, PE—Polyethylene, PET—Polyethylene Terephthalate, PP—Polypropylene, PHA—Polyhydroxyalkanoate, PLA—Poly(Lactic Acid), PBS—Poly(Butylene Succinate), PBSA—Poly(Butylene Succinate Adipate). Adapted and modified from [96].

Biodegradable polymers may be derived from fossil fuels or renewable resources, or even a blend of both (Figure 9). They usually contain ester, amide and ether functional groups. Considering the already limited fossil fuel supplies, renewable resource-based materials or bio-based polymers are preferred over conventional petroleum based products due to easy availability and superior degradability [95].

### 5.1. Fossil Fuel Based Biodegradable Polymers Used for Mulching

**Figure 10:**
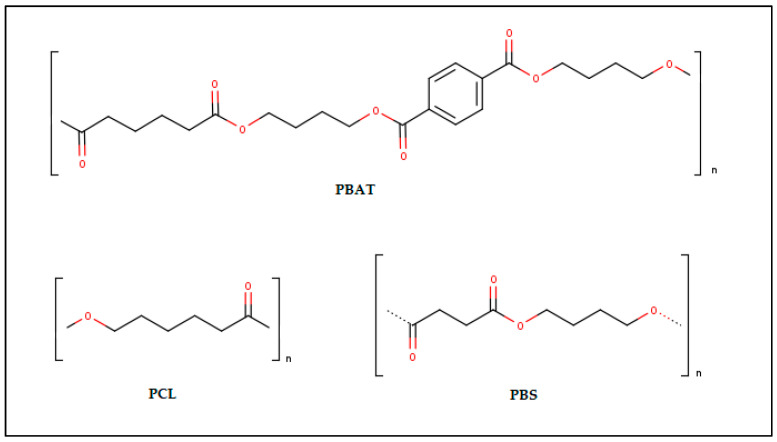
The chemical structures of fossil fuel based biodegradable polymers [98,99,100].

Biodegradable material made from synthetic polymers or derived from fossil fuels that have been used as mulch films includes polyurethanes such as polybutylene adipate terephthalate or PBAT, poly ε-caprolactone or PCL, and polybutylene succinate PBS [97]. The chemical structures of fossil fuel based biodegradable polymers is shown in Figure 10.

#### 5.1.1. Polybutylene Adipate Terephthalate

Polybutylene adipate terephthalate (PBAT) is a derivative of common petrochemicals and is considered to be fossil fuel based. It is a co-polymer of 1,4-butanediol, adipic acid and terephthalic acid. Therefore, it is an example of an aromatic–aliphatic polymer, with properties that are partially attributed to the aromatic group and partially to the aliphatic chain. It has a higher elongation break than most other polymers and is flexible [101]. PBAT also has good stretchability, impact resistance, extensibility and heat resistance, properties that are desirable for a material to be used as mulch film. The butylene adipate group imparts good soil biodegradability, making it a potential alternative to plastic mulches [102]. However, it is both expensive and highly sensitive to UV radiation. In field applications, PBAT undergoes severe crosslinking, which makes it brittle and reduces its efficacy, so it is generally recommended for short season crops only [103]. To overcome these limitations, blends of PBAT have been formulated with PLA, PPC, and/or starch. These co-polymers have exhibited increased durability and less brittleness. Additionally, they have a considerably lower cost of manufacturing since the blend is less expensive than the pure PBAT film. Now, many countries are producing PBAT-based mulch films commercially and their use is reported to have beneficial effects [104]. Blends of PBAT also have the property of functioning as controlled release systems via mulch films when fertilizers, fungicides, or herbicides are embedded into these films. The slow release of these compounds increases their efficiency and enhances crop production [2,24].

The degradability of these films may not be same in all types of soil. Studies have shown that the type of microorganisms present in the soil and the composition of soil structure influenced the extent of biodegradability of PBAT-based mulch films in field experiments [105], while more than 90% degradation of these blends under aerobic soil, within two years of application, has been documented. However, the persistence of fragments not converted to carbon dioxide or organic carbon within this time is not accounted for. There is a possibility that these micro-fragments and other chemical constituents will accumulate over time after repeated applications in the same field [106]. The degradation of PBAT under soil conditions results in the production of adipic acid and terephthalic acid, both of which are categorized as slight to moderate environmental toxins [107,108]. The presence of these compounds in the form of micro plastics has been detected in soils where PBAT-based mulch films were applied. Additionally, it was noticed that the presence of these micro plastics reduced the electrical conductivity and nitrate content in soil, which decreased the availability of water-soluble nutrients [109]. Studies have been carried out to compare the effect of mulch residues left in the soil. The results of these studies show that compared to LDPE plastic, PBAT mulch residues have a negative and harmful impact on soil bacterial community and plant growth. Further work and research need to be undertaken to ensure that PBAT-based mulch films are not hazardous to the environment, as well as the plants and microbial community of soil [110]. Since it is a petroleum-based polymer, long term use and availability of PBAT remains questionable [111].

#### 5.1.2. Poly ε-Caprolactone

Poly ε-caprolactone (PCL) is an aliphatic, synthetic, and thermoplastic polyester derived from petrochemical feedstocks. It is produced by ring-opening polymerization of ε-caprolactone, which is obtained from crude oil. It has ester linkages and can be easily broken down by microorganisms that produce the enzymes lipase or esterase [112]. The chemical structure of PCL imparts it flexibility, low melting temperature and variable viscosity. It can be moulded easily, making it a potential candidate for mulching material [113]. However, PCL mulches have poor impact, weak tear strength behaviour and there are reports on film extrusion for these mulches. It is biodegradable, but the degradation rate is slow. Therefore, it is often mixed with starch to form blends with enhanced biodegradability. The application of these blends in the field has exhibited promising results. It has been observed that these films show better degradation compared to polyethylene mulches and have a positive impact on root growth and density, which is an important indicator of plant growth [114]. Trials have also demonstrated that PCL-starch based mulch films can be degraded in most soil types and are effective in conserving soil moisture under various environmental conditions [74]. Many PCL-starch based blends are commercially available and being used as mulch films in many countries as an alternative to plastic mulches [99].

However, results of the field trials have indicated that these mulch films degrade in a shorter time if certain fungi and actinomycetes are present in the soil. In some conditions, these films degrade within a short span of 60 days, reducing their practical value and importance in agricultural practices [115]. With this in mind, this problem and the fact that the source of this polymer is non-renewable, concerns have been raised over its sustainability. Therefore, better alternatives are being searched for use in the agricultural sector [116].

#### 5.1.3. Poly Butylene Succinate

Poly Butylene Succinate (PBS) is a thermoplastic polyester which has physico-chemical properties such as conventional non-biodegradable plastic. It is made up of 1,4-butanediol and succinic acid [117]. PBS is a synthetic, petroleum-based polymer with good thermal stability and desirable mechanical properties. Its melting point is higher than other synthetic polymers but lower compared to natural polymers, so it can be melted in a shorter time and blended with other materials to develop films [118].

PBS is synthesized through various processes including co-polycondensation and reactive and physical blending, all of which impart different physical characteristics to the product formed. The higher the degree of crystallinity in the polymer, the lower its potential to be degraded by enzymes or microorganisms [119]. To overcome this problem, amorphous domains are added to the polymer by making blends with other materials. These blends have adequate strength and are available commercially as mulch films [120]. Interestingly, these PBS-based mulch films function as controlled release systems and have shown efficacy when embedded with different beneficial chemical compounds such as fertilizers or herbicides [121].

PBS and its blends are broken down by microorganisms that produce esterases. It has been found that the degradation rate of a PBS-based mulch film is influenced by the presence of certain fungi in the soil, as well as the availability of nitrogen and carbon sources to these microorganisms [122]. This may be linked to the fact that microorganisms do not produce esterases under conditions of nitrogen limitation. Similarly, the availability of carbon source plays a role in the regulation of esterase production. Therefore, soil characteristics and the type of indigenous microorganisms present are decisive factors in biodegradation of PBS-based mulch films [86]. It is important to understand that while the formulation of blends seems to be the most plausible solution to altering the properties of the polymer, PBS-based blends are often immiscible, resulting in phase separation which leads to poor mechanical properties. An alternative approach is the use of cross linkers or compatibilizer compounds in the blend, which enhance the phase mixing properties. However, the addition of these compounds compromises the biodegradability of the end-product [123]. It has been found that, compared to laboratory conditions, the biodegradation of PBS and its blends under field conditions takes longer. Low degradation of PBS-based mulches, with less than 3% degradation in more than 100 days, has been reported [124,125]. The price of PBS compared to petrochemical-based plastics is higher due to the cost of production. The large-scale production and application of PBS blends as mulch films is limited because of slow degradation, high cost and the fact that the polymer is derived from fossil fuels which are non-renewable [126].

### 5.2. Bio-Based Polymers Used for Mulching

Bio-based polymers are defined as materials formed naturally by living organisms over many growth cycles. These include lipids, proteins, and carbohydrates. Lipids are hydrocarbons, chemically, and may exist as esters, acid polyesters or free acids. They include hydrogenated fats, oils, fatty acids, and waxes [127]. Although lipids can undergo biodegradation, they are hydrophobic in nature and cannot be used as mulch films. The preparation and handling of films made of pure lipids is also very difficult, limiting their uses [128].

Proteins may be produced by microbial cells or extracted from plants and animals. These can be easily degraded in the environment by indigenous bacteria and fungi. Films made from soy protein, zein protein, wheat gluten, collagen and gelatine have been tried for mulching applications but have not been successful. The main problem associated with the use of protein-based mulches is their high-water sensitivity, which reduces the efficiency of these films [14].

**Figure 11:**
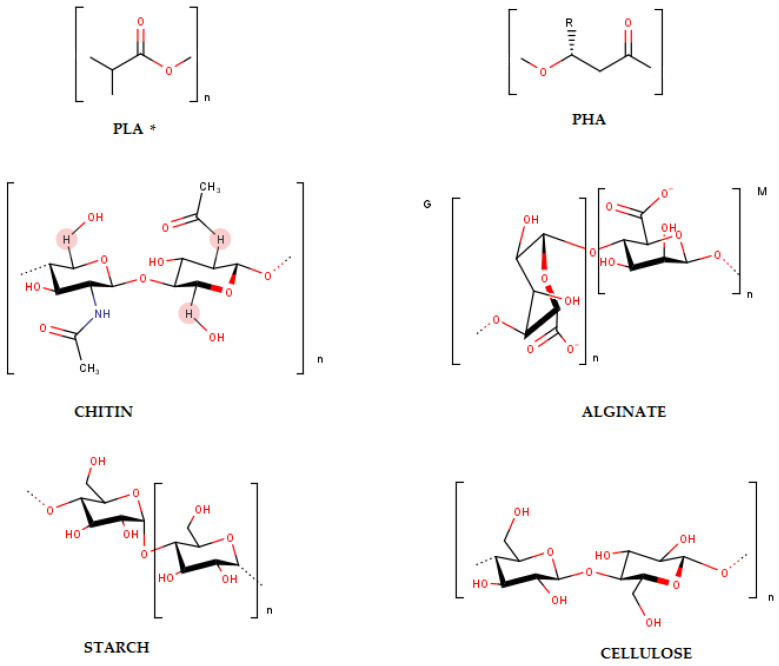
The chemical structures of bio-based biodegradable polymers (PLA* is a synthetically produced bio-based polymer, whilst PHA, Chitin, Alginate, Starch and Cellulose are naturally produced bio-based polymers) [98,99,100].

The most abundant among these polymers are carbohydrates; mainly polysaccharides. These may be produced by the bacteria or exist naturally in plants and animals. Naturally occurring polysaccharides include chitin, alginate, starch, and cellulose (Figure 11). Chemically, all of these are made up of monosaccharides that are linked together by glycosidic bonds, but the presence of various functional groups and charges impart versatility [99]. The key advantages associated with the use of these materials for mulching are enhanced biodegradability, non-toxicity, availability and low cost [127]. Bacteria can utilize carbohydrates to synthesize polymers. Two such polymers are polylactic acid and polyhydroxyalkanoates, which are chemically polyesters that are synthesized from a carbohydrate base [129]. The chemical structures of PLA and PHAs are shown in Figure 11.

#### 5.2.1. Polylactic Acid

Polylactic acid (PLA) was discovered in the 1930s and is produced by lactic acid forming microorganisms. It can be manufactured using renewable resources as the substrate for the microorganisms, such as corn, sugar beet starch and other agricultural products [129]. As suggested by the name, PLA is made from monomers of lactide which are synthesized from lactic acid. Commercially available PLA is usually a co-polymer of poly L-lactic acid and poly D-lactic acid. The monomers are produced by bacteria but the polymer is made through a synthetic process usually involving ring opening polymerization and poly-condensation. The ratio of these two polymers influences the physical properties of PLA, including its melting temperature and crystallinity, as well as molecular weight [130]. Although the large-scale production of PLA is relatively inexpensive, the total cost of the polymer is high when compared to commercially available plastics [131]. PLA has good thermoplasticity, biocompatibility and processability which allows for a wide range of applications. However, as a mulch film, brittleness is its only drawback which reduces its efficiency [132].

To counter the problems of high cost and brittleness, blends of PLA are made with other polymers. A common blend is to mix PLA with starch, which has been shown to enhance the mechanical properties of PLA. Formulation of PLA-starch blend also reduces the cost of the mulch films, making it more economical for use [133]. Studies have shown that PLA blends as mulch films are effective, biodegradable and have good mechanical properties as well as higher water holding capacity [132,134]. Additionally, PLA-based mulch films can act as controlled release systems which allow for the release of chemical compounds embedded in the film over time, to enhance plant growth while preventing leaching of these compounds. Several studies have shown their efficacy as controlled release systems when PLA-based mulch films are embedded with herbicides [2,135,136].

However, the formation of these blends is challenging and difficult. The non-compatibility of composites is an area that is still under research. It has been found that formation of unstable bubbles in the film may occur after blending, leading to wrinkles and tears in the sheet. Similarly, electrostatic attraction may result in adhesion between these films, reducing the efficacy in agricultural applications [99,137]. To overcome this problem, plasticizers are added to the blends. Plasticizers are compounds used as additives, which are added to polymers to make them softer and more pliable. However, the addition of some plasticizers to these blends is not permitted due to the stringent requirements of manufacturing materials allowed for used in agriculture. These plasticizers are not biodegradable and are released into the environment once the mulch is applied and degraded [138]. Another problem related to the use of PLA based mulch films is the commercial production of PLA using genetically modified organisms. In many European countries, as well as the USA, there are strict restrictions on the use of GMOs for manufacturing agricultural products. For example, the National Organic Program rule in the USA states that any synthetic biodegradable mulch must be produced without involvement of any GMOs, restricting the use of such products on a large-scale [139].

#### 5.2.2. Polyhydroxy Alkanoates

Polyhydroxy alkanoates (PHA) are a class of aliphatic polyesters that are produced by many bacterial species as distinct granules. Discovered in 1925, the polyesters are synthesized intracellularly and used as storage polymers for carbon sources in many prokaryotes [140]. PHA granules differ in their content and chain arrangement depending on the microorganism used for production. PHAs are polyesters of hydroxyalkanoates where the number of monomeric units may vary from as little as 600 to as many as 35,000. The presence of different functional groups on the monomeric units is used as a classification system for types of PHAs [141]. The carbon source utilized for production, constituents of the media, fermentation process and conditions, as well as the downstream processes used for purification are other factors that have an effect on the structure and composition of the polymer [142,143,144]. More than 150 different PHAs are known to date, based on the combination of the monomeric units that make up the chain. The most common and well-known PHAs are poly 3-hydroxybutyrate(PHB) and poly 3-hydroxybutyrate-co-3-hydroxyvalerate (PHBV). Both are short chain polymers and available commercially [145].

Generally, the mechanical properties of PHAs do not support their use as mulch films. However, blending different PHAs together or with other polymers improves their mechanical strength. The main feature of PHAs that imparts an advantage is their ability to function as controlled release systems for agricultural chemicals. Studies have shown that PHB blends are particularly effective as mulch films for the slow and controlled release of pesticides and fungicides [146,147]. Blends of PHB with PLA have desirable mechanical properties such as polyethylene mulch films [91]. Biodegradation of PHB blends has been studied in a wide range of conditions including a controlled laboratory environment as well as natural habitats including different types of soil, river water, activated sludge, and compost. PHB is known to degrade rapidly in both aerobic and anaerobic environments, and thus can be disposed of easily without any negative impact on the environment [148,149].

The desirable characteristics such as structural variability, raw material availability, and biodegradability of PHB polymers make them an ideal candidate for replacing plastic as mulch films. Yet, despite the microbial source and versatility, the large-scale production of PHAs is restricted by the high cost of the manufacturing process [14]. Different production strategies, including the use of waste material as a carbon source, have been employed to decrease the cost of production. While these studies have shown positive results, the extraction of PHAs from bacterial cells still contributes significantly to the higher cost of the process. Extensive chemical extraction methods cause degradation of the polymer during the purification process. Therefore, efficient biological methods for extraction of PHAs are being investigated to decrease the cost of the production and to ensure that the useful properties of the polymer are not lost [150].

#### 5.2.3. Chitin

Chitin is found naturally in the cell walls of fungi and yeast, and in the exoskeleton of arthropods. The most abundant crystalline form of the polymer is α-chitin, which may be obtained from seafood waste in large quantities [151]. It is made up of D-glucosamine and N-acetyl-D-glucosamine residues which are linked together. The deacetylation of chitin forms chitosan. Chitosan readily dissolves in dilute acids but remains insoluble in water. It can also be moulded to form films. These two features make it a good choice for use as a material from which to make mulch films [152]. Chitosan is the second largest and abundant biological polysaccharide found in nature. However, films of pure chitosan may be brittle due to low fluidity of the linear chain. Plasticizers such as glycerol may be added to chitosan to enhance its flexibility and improve fluidity [153].

When used for mulching, chitosan has exhibited the potential to control weed growth much better compared to some herbicides [154]. It can also function effectively as a controlled release system and chitosan mulch films may be loaded with fungicides to protect plants against fungal diseases [155]. Chitosan is also considered to promote plant growth by contributing to soil nutrients, and many studies have shown that it enhances the growth rate, number of flowers and quality of vegetables when used as a mulch for various plant species [156,157,158]. However, it has been observed that the use of chitosan-based mulch affects soil temperature, increasing it during the day and lowering it at night, which has an adverse impact on plant growth [159]. Moreover, the production of chitosan from chitin is an expensive process. The interference in soil temperature and high cost mean that chitin and chitosan are usually not preferred as a mulching material [14,152].

#### 5.2.4. Alginate

Alginate is an aliphatic, water-soluble polymer found in the cell wall of brown algae. It is chemically made up of β-d-mannuronic acid (monomer M), and α-l-guluronic acid (Monomer G). The sequential arrangement and proportions of these two monomers in the alginate chain impart its different properties [160]. It can form three-dimensional mesh networks in the presence of cations, where the cation acts as a cross-linker that joins the alginate chains from the G residues. This property has been exploited for the production of gels or films from alginate, mainly with calcium ions, for the controlled release of active agents [161]. Alginate mulches have been produced as a solution of sodium alginate which is sprayed on the soil, where it forms cross-linking with naturally occurring calcium ions. Once the water has evaporated, a thin layer of the polymer appears on the soil surface that functions as a mulch film [162]. Field trials have shown that these alginate mulches improve plant growth and have a positive impact on the population of soil microorganisms including fungi and actinomycetes. It also lowers soil temperature, making it a good option for use in hot climatic zones [163]. Alginate is known to act as a biostimulant and promotes plant growth, specifically through better root development and fruit quality, and it also improves the plant’s ability to tolerate salt stress. Therefore, the use of alginate mulches may contribute in many ways to enhanced plant growth [164,165,166]. Moreover, alginate is non-toxic and biodegradable, and has good water holding capacity [167].

However, the main problem with the use of alginate-based mulch films is their dependency of synthesis on the presence of calcium ions in the soil, since the films cannot be produced in the absence of these ions. Furthermore, the stiff structure of sodium alginate and the rigidity of the resulting cross-linked film leads to rips and tears in the mulch film, which paves way for the growth of weeds [14]. A method to overcome this problem is the formation of blends including polyglycerol or hydroxyethylcellulose. While these blends have shown potential to be used as mulch films, the cost of production increases considerably, reducing the overall efficacy of the product [168].

#### 5.2.5. Starch

Starch is the main storage polysaccharide found in plants and is made up of the chains of amylose and amylopectin. Amylose is linear and consists of glucose units linked by α-1, 4 glycosidic linkages, while amylopectin has branches that stem out of the main chain. The main chain is made up of glucose units joined by α-1, 4 glycosidic linkages while the branch points have α-1, 6 glycosidic linkages [169]. Starch is found in the form of granules in seeds, roots, and tubers. Depending upon the plant source, starch may consist of amylose, amylopectin and other compounds such as lipids, proteins and phosphate or ester groups in minor quantities. The proportion of amylose and amylopectin influences the properties of starch. High amylose starches produce stiffer gels and stronger films, while high amylopectin starches produce softer gels which are more stable over longer periods of time. The proportion of these components is the main factor that helps to choose the source of starch and its applicability [170]. Commercially, starch is extracted from corn, wheat, rice, sorghum and potato depending on the geographical regions and local availability [171].

Starch is one of the most abundant and cheap polymers that can be used to replace plastic. It can be easily processed without any stringent requirements and can be produced commercially using already installed plastic processing machinery/equipment [172]. Considering these attributes and the fact that it can be used easily to form a film through the process of gelatinization, it is a good choice for use as a mulching material [173]. However, it has poor water resistance or high hydrophilicity which leads to enhanced degradation. In fact, it has been observed that more than 33% of the starch mulch film degrades within 55 days of application as a mulching material [64]. Starch films also have low elastic strength and are brittle, which means that they often tear apart while being spread on the field [68]. One way to overcome these problems is to modify or blend starch with other materials such as glycol, chitosan, and PLA. Films synthesized in this manner have exhibited better elastic strength but are expensive to produce and are often sensitive to high humidity. Moreover, the mechanical properties of these films are still not comparable to plastic mulch films, limiting their potential use in agriculture [174,175].

#### 5.2.6. Cellulose

Cellulose is the main structural component of plant cell walls and is the most abundant polysaccharide in nature. It is a linear homopolymer made up of D-glucose units joined through β- 1, 4 glycosidic bonds [176]. Cellulose is found in plants and can also be produced by a number of microorganisms including common soil-borne bacteria. Naturally occurring cellulose is found in two crystalline or allomorphic forms, Iα and Iβ, depending upon the network of hydrogen bonds formed between the hydroxyl groups of cellulose chains. Iα is predominantly found in plants, while Iβ is the form of cellulose produced by bacteria [177]. These allomorphs vary in their solubility because of the hydrogen bonding pattern. Bacterial cellulose is preferred over plant cellulose due to relative abundance and ease of production and extraction [178]. Additionally, bacterial cellulose has good biodegradability, purity, water holding capacity, transparency, flexibility, and greater mechanical strength, making it an ideal material to replace plastic for mulch films [179]. Field trials have demonstrated that mulch films made of bacterial cellulose better retain soil moisture and are effective in providing a suitable microclimate for plant growth. Furthermore, these films can be modified as composite films to release nutrients by the addition of fertilizers [180,181].

The main challenge for the production of cellulose on a commercial scale is the higher cost of this process. Scientists are investigating the use of cheap waste material as a substrate for the bacteria to produce cellulose to decrease the cost of production [182,183]. Other aspects to reduce production costs include developing new strategies for agitated and static culturing and designing new, cost-effective fermentation vessels [184].

**Figure 12:**
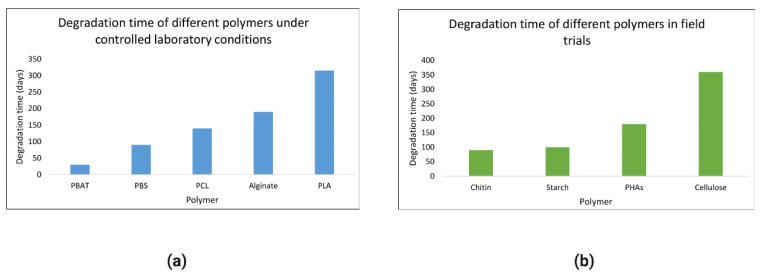
The degradation time estimated for different biodegradable polymers (**a**) under controlled conditions and (**b**) in field trials.

A comparison of the advantages and disadvantages of biodegradable polymers and their degradation times is given in Table 2 [103,104,105,106,107,108,109,110,111,112,113,114,115,116,117,118,119,120,121,122,123,124,125,126,127,128,129,130,131,132,133,134,135,136,137,138,139,140,141,142,143,144,145,146,147,148,149,150,151,152,153,154,155,156,157,158,159,160,161,162,163,164,165,166,167,168,169,170,171,172,173,174,175,176,177,178,179,180,181,182,183,184] and Figure 12, respectively [185,186,187,188,189].

## 6. Commercially Available Biodegradable Mulches—Current Trends and Challenges

Despite the challenges and problems related to production of biodegradable mulch films, consistent research efforts over a number of years have resulted in the development of some commercial mulching materials. All these mulch films are polysaccharide-based and are manufactured by blending two or more polymers [99].

The first biodegradable mulch film used in agriculture was Mater-Bi^®^, which was produced by Novamont, an Italian company. The mulch film is made of a blend of poly ε-caprolactone and thermoplastic starch and possesses reasonable biodegradability and mechanical strength [82]. Its application in the field has demonstrated that the material can be used effectively for mulching purposes [190]. Other commercially available mulch films include Biomax^®^ TPS, Biopar^®^, Bionelle, Biosafe™, Ingeo^®^, and WeedGuardPlus [65,191,192]. These films are manufactured by various companies in different countries and are being used in farming practices (Table 3).

Results obtained from field experiments have indicated that biodegradable mulch films are as effective as polyethylene mulch in controlling weed infestation and promoting plant growth. However, the increasing demand for food and cash crops has led to a continuous rise in the global consumption of plastic mulch film. Although there is a wide range of biodegradable material available on the market, the quantity is still not sufficient to completely replace non-degradable plastic mulch films [202]. The main challenge associated with the large-scale production of biodegradable mulch films is the economic viability of the production process. The profit and economic feasibility analysis of biodegradable mulches indicates that if these are to be used as alternatives to plastic mulches, governments need to provide subsidies to promote their use through extensive marketing [203,204,205].

## 7. Future Prospects of Biodegradable Mulch Films

Despite extensive research in the development of biodegradable mulch films commercially, the large-scale use of these materials is still limited. The biodegradable mulching material needs to be cost effective and easily accessible to the farmers for complete replacement of polyethylene in the field. Commercially available biodegradable mulch films are not preferred by most farmers because these are expensive, difficult to manage and require specialized equipment for application [206]. This means that although some alternatives to plastic mulches are available, they are not being used. Governments need to play a part in subsidising these mulch films and raising awareness about their benefits over plastic mulch films. For example, some regional authorities in Spain are giving incentives to farmers who use biodegradable mulch films to promote their use [204]. Such policies need to be adopted globally to encourage the use of biodegradable materials.

There is a need to develop polymers suitable for mulching in different climatic zones, with a wide range of temperatures and soil types, so that these can be utilized for the production of different crops [207]. It is currently a challenge to develop a material that fits the criteria of mulching products with good physical characteristics, durability, and biodegradability. In this context, while there are standard testing methods for determination of mechanical strength, biodegradation analysis is still raising questions on many platforms. The biodegradability testing methods have several shortcomings, since these are mostly carried out in laboratory conditions and rely on indirect testing methods without taking soil characteristics into account. It is important to understand that the biodegradation process in the field is affected by many factors and the polymer may not exhibit the same rate of breakdown in lab and field conditions [208]. Even if the polymer degrades in soil, the long-term effects of any residual matter left after biodegradation of these polymers is yet to be investigated. Therefore, there is a dire need to develop testing methods that can estimate the biodegradation of the polymer in field conditions and allow for a close-to-reality simulation of the possible residual effects [139].

Among all polymers that may be used, cellulose offers potential for further research since the only problem associated with it is the cost of production. New processing methodologies such as 3D printing and electrospinning are also being considered for production of mulch material and can be used for lowering the cost of cellulose [209]. However, work in this area is still limited to laboratory trials, and field application still needs to be carried out to commercialize these products for further use [86].

## 8. Conclusions

This review provides a detailed account of the history of mulching, the concerns about the use of plastic mulch films and their impact on the environment. It also discusses the alternative biodegradable polymers with the potential to be used for mulching, including those produced naturally by microorganisms, animals and plants as well as some derived from fossil fuels, alongside their advantages and limitations. Most of the biodegradable polymers have characteristics that are a compromise in terms of parameters that need to be met for agricultural use in terms of maintaining the optimum microclimate for plant growth for the whole growth cycle and sufficient high tensile strength. For example, materials that exhibit good biodegradation often lack mechanical strength, and vice versa. The formation of blends or use of co-polymers appears to be a promising solution but presents a limitation of increasing the cost of production significantly. The future outlook for the development of biodegradable mulch films is nonetheless favourable. The cost of production can be reduced by adopting waste valorisation strategies wherever possible, and advanced scientific techniques can be used to improve the quality of the polymer produced so that it can meet the required standards. The discussion reflects the potential for research and development in the design of biodegradable mulch films. Further research in all domains of production, design, characterization, biodegradation analysis and commercialization needs to be carried out to encourage the use of biodegradable polymers for mulching to promote sustainable practices.

## Figures and Tables

**Table 1:** Comparison of the sources, advantages and disadvantages of organic and synthetic mulch films.

Type of Mulch	Source	Advantages	Disadvantages
Organic	Compost	Adds nutrients to soil	Promotes pests and disease-causing organisms
	Straw and Husks	Inexpensive with long life	Harbours pests and weed seeds
	Sawdust	Readily available	Hardens over time and does not allow water to seep into the soil
	Grass clippings	Controls weeds effectively	Develops own root systems and competes with plant for nutrients
	Paper clippings	Decomposes easily	Rips and tears apart during application
	Bark chips and Pine needles	Retains soil moisture	Reduces soil pH
Inorganic	Glass pieces	Aesthetically appealing	Interferes with soil temperature and does not allow sunlight to penetrate
	Rubber clippings	Effective recycling	Hazard of fire and may promote zinc toxicity in plants
	Plastic	Retains moisture and controls weed growth	Labour intensive and contributes to plastic pollution
	PAC plastic	Retains moisture, controls weed growth and degrades in presence of UV light	Forms micro plastics in soil

**Table 2:** Comparison of the advantages and disadvantages of different synthetic and bio-based biodegradable mulch films.

Type of Biodegradable Polymer	Polymer	Advantages	Disadvantages
Synthetic	Polybutylene adipate terephthalate	Good impact resistance and extensibility	Produces microplastics
	Poly ε-caprolactone	Flexible material and effective in retaining soil moisture	Degrades very quickly and has to be replenished frequently
	Poly Butylene Succinate	Good thermal stability	Expensive with limited biodegradability
Bio-based	Polylactic acid	Good processibility and thermoplasticity	Brittle and expensive
	Polyhydroxyalkanoates	Can act as controlled release system	Expensive to produce and lacks mechanical strength
	Chitin	Controls weed growth	Alters soil temperature and expensive to produce
	Alginate	Acts as biostimulant and promotes plant growth	Rips after application
	Starch	Abundant and cheap	Brittle and low tensile strength so tears apart during application
	Cellulose	Flexible with good tensile strength	Expensive to produce on large-scale

**Table 3:** Some commercially available mulch films and the crops grown using them.

Commercial Mulch Film	Composition	Manufacturer	Crops Grown Using Mulch Films	Reference
Biomax^®^ TPS	TPS + starch	DuPont	Corn	[193]
Bionolle	TPS + PLA + (PBS or PBSA)	Showa Denko Europe	Onion	[194]
Mater-Bi^®^	TPS + PCL	Novamont	Tomato	[195]
Eco-Flex^®^	Starch + PBAT	BASF	Strawberry, Tomato	[196,197]
Ecovio^®^	TPS + PBAT + PLA	BASF	Cucumber	[198]
EcoWorks	TPS + PBAT	Cortec Corporation	Bell Pepper	[199]
Ingeo^®^	Starch + PLA	Nature Works	Tomato	[200]
Naturecycle	Starch + polyester	Custom Bioplastics	Pumpkin	[106]
WeedGuardPlus	Cellulose	Sunshine Paper	Strawberry	[201]

## Data Availability

Not applicable.
